# Utilisation of Intermediate Care Units: A Systematic Review

**DOI:** 10.1155/2017/8038460

**Published:** 2017-07-09

**Authors:** Joost D. J. Plate, Luke P. H. Leenen, Marijn Houwert, Falco Hietbrink

**Affiliations:** Division of Surgery, University Medical Centre Utrecht, Utrecht, Netherlands

## Abstract

*Background. *The diversity in formats of Intermediate Care Units (IMCUs) makes it difficult to compare data from different settings. The purpose of this article was to describe and quantify these different formations and utilisation.* Methods.* We performed a systematic review extracting geographic location, nomenclature used, admitting specialties, open (admitting specialist in charge) or closed (intensivist/generalist in charge) management format, location in hospital, number of beds, nursing workload, medical staff to patient ratios, and modalities—possibilities and limitations—implemented.* Results.* Nomenclature used was High Dependency Unit (56.8%) or Intermediate Care Unit (24.3%), with the latter one increasingly being used recently. The median number of beds was 6 (IQR 4–10). Location (*p* < 0.001) and admitting specialties (*p* = 0.03) were related to the management format. IMCUs integrated or adjacent to Intensive Care Units were more often capable of using single vasoactive medication (*p* = 0.025). The mean nurse to patient ratio was 1 to 2.5.* Conclusions.* IMCUs often have a specific task in a hospital, which is reflected in location, format, and utilisation. The management format depends on location and admitting specialist while incorporated supportive treatment modules reflect its function. Common IMCU denominators are continuous monitoring and respiratory support, without mechanical ventilation and multiple vasoactive medications.

## 1. Background

An Intermediate Care Unit (IMCU) is logistically situated between the Intensive Care Unit (ICU) and the general ward. It can function as a physically independent unit or as a dedicated section, incorporated within the ICU [1–3]. It can act as a “step-up” or “step-down” unit between the general ward and the ICU [3–5] but can also be used to admit patients from the Emergency Department or Recovery ward [5, 6]. From a historical perspective, most IMCUs originated from specific medical specialties or were introduced for a specific function (i.e., obstetric care, cardiac care), while later adding function and scope [7]. The characteristics, type, and amount of services provided depend on factors such as resource availability, institutional infrastructure, and the overall health care system [8].

Positive effects of IMCUs include generating extra ICU capacity by earlier discharge of some ICU patients as well as an alternative to ICU admission for patients who only require intensive monitoring, specific support, or procedures [9]. Recently, a significantly reduced mortality was observed in patients admitted to an ICU in hospitals with an IMCU compared to hospitals without an IMCU [7].

In addition, due to the buffer function of the IMCU, the duration of ICU admission can be reduced and it seems reasonable that the lower need for ICU beds decreases health care costs. However, there is relatively little data published to support this benefit [1]. This lack of data might be attributed to the heterogeneity between IMCUs and the lack of a clear common denominator as to what determines an IMCU and how it can be formatted. Although differences in setting and utilisation make it difficult to compare data, comparison of different utilisation of IMCUs is needed in order to establish the best systems design, optimize critical care capacity, and manage health care costs [1, 3].

The primary aim of this systematic review was to describe and quantify the formation and utilisation of IMCUs. By providing an overview of the possible formats, supportive possibilities, and limitations we sought to clarify and define the IMCU and to determine its common characteristics.

## 2. Material and Methods

### 2.1. Information Sources

The nomenclature of the IMCU consists of various names [11], such as Intermediate Care Unit, step-down unit, High Dependency Unit, Progressive Care Unit, Medium Care Unit, High Care Unit, Transitional Care Unit, Special Care Unit, Subintensive Care Unit, Semi-Intensive Care Unit, and many synonyms of these. We chose the term Intermediate Care Unit in this article since we regard this as being the most appropriate due to the location of the unit (intermediate) between the general ward and the ICU.

We performed a comprehensive literature search in multiple electronic databases (Medline, Embase, and Cochrane), where we searched all publications up to 10.09.2016. The search we constructed used the following keywords in title/abstract search: “Medium Care Unit” OR “Intermediate Care Unit” OR “High Care Unit” OR “High Dependency Unit” OR “Progressive Care Unit” OR “Step up unit” OR “Step down unit” OR “Transitional Care Unit” and synonyms of those [see Supplementary Material 1 for all search terms in Supplementary Material available online at https://doi.org/10.1155/2017/8038460]. We performed a cross-reference check of the articles in full-text review.

### 2.2. Study Selection

The following are inclusion criteria for full-text review and data abstraction we used: (1) publication in English or Dutch and (2) description of an IMCU. We excluded articles about cardiac, obstetric, gynaecological, paediatric, and psychiatric care units due to their specific small spectrum model of care for well-defined disease entities. This stands in contrast to the possibility to harbour different patient groups and diseases in IMCUs such as surgical, general medical, or neurological IMCUs. We excluded articles describing a Transitional Care Unit between hospital and nursing homes, since these were not comparable to the IMCUs situated between ICU and ward. We also excluded case reports, conference abstracts, and reviews if we were unable to extract published data per IMCU.

For the outcome, description of an IMCU, we included articles describing anything about their admitting specialties, management format, location in the hospital, nursing workload, supportive possibilities, or limitations. Of the latter, it should be noted that respiratory support was defined as the possibility or limitation to deliver supplementary (though not high-flow) oxygen. Mechanical (invasive) ventilation was noted separately.

To describe the nursing workload, we used the nurse to patient ratio, Therapeutic Intervention Scoring System (TISS-78) [12], with its simplified version TISS-28 [13], and the Nursing Activities Scores (NAS) [14]. The TISS and NAS, originally created for the ICU, are both measures for the nursing workload. The TISS-28 represents 10.6 minutes of working time per nursing shift for each point and the NAS represents the mean percentage of working time spent on a patient per nursing shift [5, 13].

### 2.3. Data Extraction

From each of the included articles, we extracted the following data: name given to the IMCU, country of the IMCU, medical specialties being admitted, management format used (open or closed), the location of the IMCU (integrated unit in the ICU, independent but adjacent to an ICU, separate independent unit, or part of the ward), number of beds, nursing workload (including nurse to patient ratio and TISS-28, TISS-78, and NAS), medical staff to patient ratio, and supportive possibilities and limitations. The medical staff was extracted at resident, registrar, and consultant level.

### 2.4. Data Synthesis and Analysis

If multiple articles described the same unit but differed regarding one of the studied variables, we chose to report the information of the most recent article. If an article described more than one unit, namely, before and after changing its structure, we included both units in our analysis. However, in such cases we used article specific items (name and country) only once per IMCU. For the nurse to patient ratio, we calculated the average if the article reported a range of possible nurse to patient ratios in their IMCU. If the article reported the TISS-78 score, we calculated the TISS-28 using the following equation: TISS-28 equals 3.33 plus 0.97 times the TISS-78 score [13].

To assess for normality of the continuous variables, we used graphical visualisation and Shapiro-Wilk Normality tests. Since the continuous variables were not normally distributed, we chose to report the median with interquartile range. We analysed differences in continuous variables using the Mann-Whitney *U* test or Kruskal-Wallis *H* tests. To compare the categorical variables, we used Fisher's exact tests since in all cases more than 20% of expected values were below 5. We considered a *p* value of less than 0.05 to be statistically significant. We performed all statistics using R software for statistical computing version 3.3.2. [15] with the additional packages “ggplot2” [16], “ggmap” [17], and “reshape” [18].

## 3. Results

### 3.1. Study Selection

**Figure 1:**
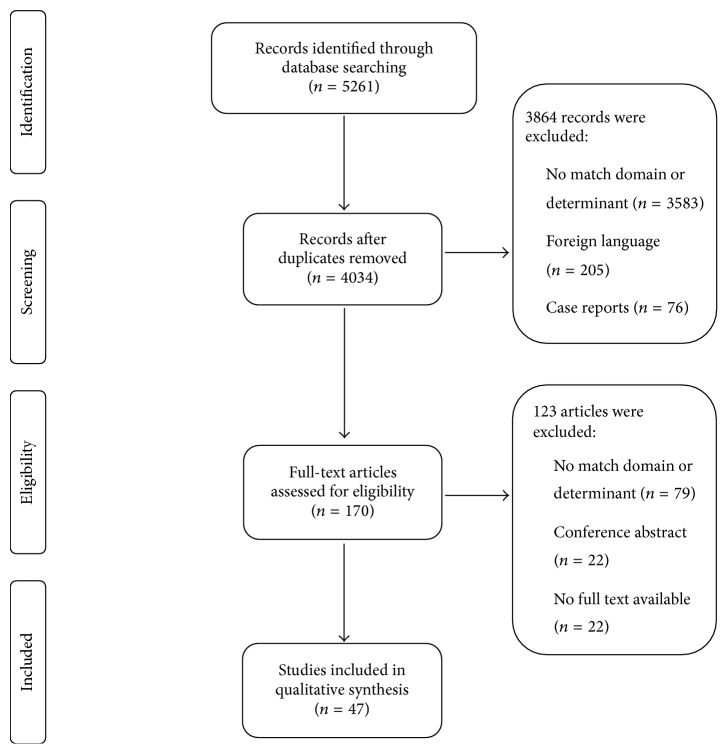
Preferred Reporting Items for Systematic Reviews and Meta-Analyses (PRISMA) flow diagram for study selection [10].

From 4034 titles and abstracts, we selected 148 articles for full-text review (Figure 1). Of these, we included 47 studies of 39 IMCUs in 11 countries.

### 3.2. Study Characteristics

A summary of the characteristics of the included studies and their corresponding IMCUs are described in Table 1 [see Supplementary Material 2 for the full study characteristics per IMCU]. Seven units were described in more than one article, while two articles described their IMCU before and after changes in management or location structure [19, 20].

**Figure 2:**
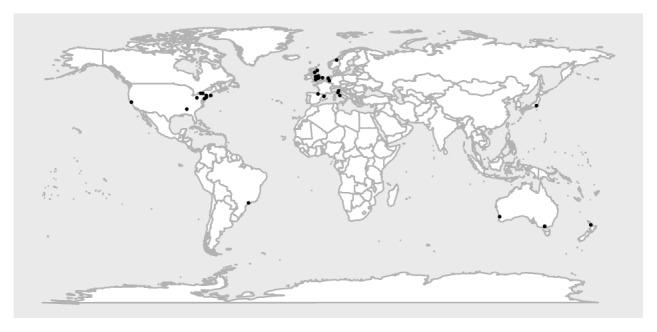
Distribution of reported Intermediate Care Units around the world. This world map demonstrates the location of Intermediate Care Units as reported by our included studies. Most reported Intermediate Care Units are situated in Europe and around the east coast of the United States.

The included articles date from 1983 to present and were mainly from Europe (*n* = 24, 64.8%) and most frequently from the United Kingdom (*n* = 16, 43.2%). The rest of the articles were from the USA (*n* = 6), Australia (*n* = 3), Canada (*n* = 1), New Zealand (*n* = 1), Japan (*n* = 1), and Brazil (*n* = 1). This distribution of reported IMCUs is depicted in Figure 2.

### 3.3. Nomenclature

The included articles used the term High Dependency Unit (*n* = 21, 56.8%), Intermediate Care Unit (*n* = 9), Step Down Unit (*n* = 4), Progressive Care Unit (*n* = 2), and Medium Care Unit (*n* = 1). Of the articles using the term High Dependency Unit, 90.5% originated from one of the Anglo-Saxon countries (UK, Australia, and New Zealand). This term was not at all used in articles from the USA. The term Medium Care Unit was only used in one Dutch article [5].

The use of the term High Dependency Unit has decreased from 90.91% (*n* = 10) before 2001 (*n* = 11 articles) to 36.4% (*n* = 4) after 2010 (*n* = 11 articles). Consequently, the term Intermediate Care Unit has increased in frequency from 0% (*n* = 0) before 2001 to 45.5% (*n* = 5) after 2010.

### 3.4. Specialties Using the IMCU

In 94.9% (*n* = 37), the admitting specialties were reported. A total of 51.4% IMCUs (*n* = 19) treated only surgical patients, while 35.1% (*n* = 13) treated both surgical and medical patients, 10.8% (*n* = 4) treated only medical patients, and 2.7% (*n* = 1) treated emergency patients of surgical and medical specialties. Thus, surgical patients were treated in a total of 89.2% (*n* = 33) of the IMCUs.

Of the IMCUs admitting only surgical patients, 63.2% (*n* = 12) provided care for multiple surgical specialties, 21.1% (*n* = 4) admitted only postoperative patients, and 15.8% (*n* = 3) admitted only patients from a single surgical specialty. Of the latter group, 2 IMCUs specifically treated thoracic surgical patients only [51, 57] and 1 IMCU specifically treated otolaryngology and maxillofacial patients [22].

### 3.5. Management Format

The management format was reported in 76.9% (*n* = 30) of all articles. In total, 56.7% had an open format IMCU, in which the attending specialist remains in charge during admission at the IMCU. The other 43.3% had a closed format IMCU, with a designated specialist (usually an intensivist, but occasionally a hospitalist or emergency physician) in charge of the IMCU.

IMCUs with an open format often only treated surgical patients (*n* = 12, 70.6%), while IMCUs with a closed format often treated both surgical and medical patients (*n* = 8, 61.5%) (*p* = 0.03).

### 3.6. Location

Of a total of 27 IMCUs (69.2%) the location was reported. Of these, 4 IMCUs were integrated in the ICU, while 10 IMCUs were independent, but adjacent to the ICU. In total, 11 IMCUs were independent, separate units and 2 units were part of the hospital ward.

Only 1 of the IMCUs integrated in the ICU had an open format. All the independent but adjacent IMCUs (*n* = 8) had a closed format, most of the independent and separate IMCUs (*n* = 9) had an open format, and all the IMCUs as part of the hospital ward (*n* = 2) had an open format (*p* < 0.001).

**Figure 3:**
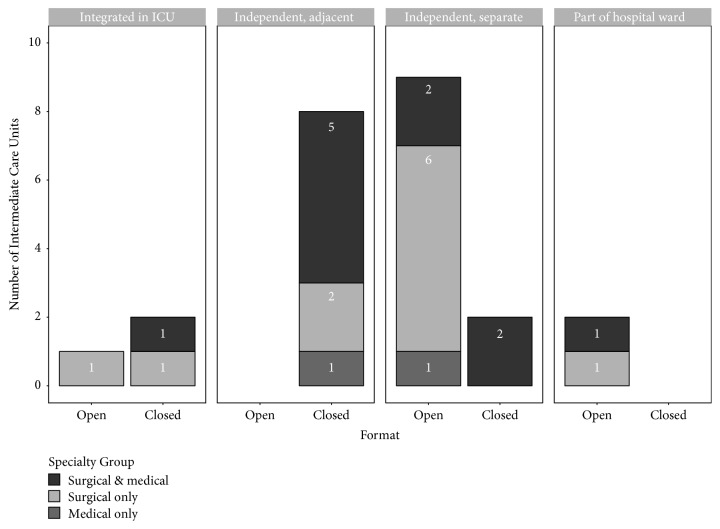
Number of Intermediate Care Units per location, format, and admitting specialties. This chart shows that most of the Intermediate Care Units were either (1) closed format, adjacent to the Intensive Care Unit and treating surgical and medical patients, or (2) open format, independently located and treating surgical patients only.

There was no relationship between location of the IMCU and the admitting specialty (*p* = 0.69). The relationship between location, format, and admitting specialties is shown in Figure 3. The admitting specialties, format, and location did not significantly differ over time.

### 3.7. Number of IMCU Beds

The number of beds was reported for 82.1% of the IMCUs (*n* = 32). The number of IMCU beds ranged from 2 to 24 with a median of 6 and an interquartile range of 4 to 10. The median numbers of beds were 8 in an IMCU integrated in an ICU (*n* = 4) (IQR 6.8–8.3), 6 (IQR 4–8.8) in an independent, adjacent IMCU (*n* = 10), 9 (IQR 6–11) in a separate independent IMCU (*n* = 9), and 2 in an IMCU as part of a hospital ward (*n* = 1; *p* = 0.20).

In an open format IMCU, the median number of beds was 6 (IQR 4–7). In a closed format IMCU, the median number of beds was 8 (IQR 5.5–9.3) (*p* = 0.31).

### 3.8. Nursing Workload

The median nurse to patient ratio at IMCUs was 1 : 2.5 (IQR 2–3.5), based on 24 IMCUs (61.5%). This ratio did not significantly differ per location or format of IMCU. The TISS-28 (*n* = 3), TISS-76 (*n* = 1), and NAS (*n* = 1) were reported for only a few IMCUs (*n* = 5). The reported range of the mean TISS-28 was 5.8 to 19.8 (1.02–3.50 hours of work per patient per nursing shift) [20, 37, 58, 64]. The mean NAS was reported once and varied from 37.0 to 44.3, differing per nursing shift [5].

### 3.9. Medical Staff

Details about the medical staff at the IMCU were reported in 11 articles (28.21%). Of these, 6 articles (15.38%) reported having residents at the IMCU, 3 (7.69%) reported having registrars, and 10 (25.64%) reported having consultants in charge. Information about the number of medical staff and the derived patients per medical staff ratio was reported in 7 articles (17.95%). From this it followed that the median number of patients was 8 (IQR 7–9, *n* = 3) per resident, 9.5 (IQR 6.75–12.25, *n* = 2) per registrar, and 9 (IQR 6–10, *n* = 6) per consultant. Of the two studies which reported having a registrar at the IMCU, one also reported having a consultant available while the other one did not provide any more information. One study reported having two residents, while all other studies reported having a maximum of one consultant and/or one resident.

### 3.10. Possibilities and Limitations

Studies only scarcely reported the possibilities and limitations of their IMCUs, leading to missing information ranging from 11 (28.2%) missing values for haemodynamic monitoring to 38 (97.4%) missing values on sedative use and specific interventions.

**Figure 4:**
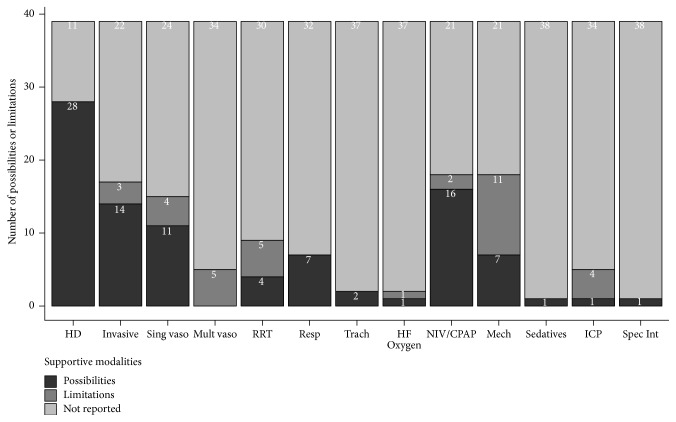
Possibilities and limitations of Intermediate Care Units. This chart shows that Intermediate Care Units always provided haemodynamic monitoring and respiratory support, while common limitations were mechanical ventilation, multiple vasoactive medications, renal replacement therapy, and intracranial pressure management. HD = hemodynamic monitoring; Invasive = invasive monitoring; Sing vaso = single vasoactive medication; Mult vaso = multiple vasoactive medications; RRT = renal replacement therapy; Resp = respiratory support; Trach = tracheostomy care; HF Oxygen = high-flow oxygen therapy; NIV/CPAP = noninvasive ventilation/continuous positive airway pressure; Mech = mechanical ventilation (ventilation of weaning); ICP = intracranial pressure measurement; Spec Int = specific interventions.

Reported supportive possibilities of IMCUs (Figure 4) were hemodynamic monitoring (*n* = 29), invasive monitoring (*n* = 14), single vasoactive medication use (*n* = 12), renal replacement therapy (*n* = 4), respiratory support (*n* = 7), tracheostomy care (*n* = 2), noninvasive ventilation or continuous positive airway pressure (NIV/CPAP, *n* = 16), high-flow oxygen therapy (*n* = 1), mechanical ventilation in otherwise stable patient (weaning or postoperatively, *n* = 7), the continuous use of propofol as a sedative (*n* = 1), intracranial pressure measurement (*n* = 1), and specific interventions, for example, chest tube placement and thrombolysis (*n* = 1).

Reported limitations of supportive care at IMCUs were mainly mechanical ventilation (*n* = 11), renal replacement therapy (*n* = 5), single vasoactive medication use (*n* = 4), multiple vasoactive medication (*n* = 5), and intracranial pressure (*n* = 4). Other reported limitations were invasive monitoring (*n* = 3), NIV/CPAP (*n* = 2), or high-flow oxygen therapy (*n* = 1).

Despite the large amount of missing data, there was a relationship between location and single vasoactive medication use (*p* = 0.025). All IMCUs integrated in the ICU (*n* = 3) or adjacent to the ICU (*n* = 3) reported single vasoactive medication as a possibility at their unit. This was in contrast with separate, independent IMCUs, of which all (*n* = 3) reported single vasoactive medication use as a limitation.

## 4. Discussion

IMCUs were mainly called Intermediate Care Units or High Dependency Units and most reports originated from Europe and the east coast of the United States. They predominantly treated surgical patients (89.2%). Sometimes this was combined with medical patients (35.1% of total). Their format was either open (56.7%) with the attending specialist in charge or closed (43.3%) with the intensivist in charge. The location of IMCUs was mostly adjacent to the ICU (37.0%) or in a separate location (40.7%). The number of beds ranged from 2 to 24 with a median of 6. The median nurse to patient ratio was 1 to 2.5. Medical staff generally consisted of one consultant and one resident or registrar for on average, respectively, 8 or 9.5 IMCU patients.

IMCU care always included haemodynamic monitoring and respiratory support as supportive monitoring or treatment modules. Other modalities which could be incorporated into an IMCU to meet hospital-specific needs varied widely. Also, due to these specific needs and corresponding functions each specific IMCU had its own limitations. However, since unstable respiratory patients could not be admitted at any IMCU, it appears that respiratory instability is a limiting factor for admission. This could probably be due to the lack of mechanical ventilation; the only IMCUs providing mechanical ventilation only did so in patients weaning after ICU admission or surgery. Also, some IMCUs integrated or adjacent to ICUs offered the possibility for single vasoactive medication use, whereas this was a reported limitation at separate, independent IMCUs.

We found that there is a growing trend towards using the term Intermediate Care Unit, which is probably due to the logistic position of the IMCU in the hospital. Although we argue this term is most suitable, we should be aware that this term is also occasionally used for long-term outpatient care or rehabilitation centres. Furthermore, we found strong evidence for an association between format and both location and admitting specialties. IMCUs located adjacent to the ICU always had a closed format and treated both medical and surgical patients. Those located separately often had an open format and treated only surgical patients.

This was the first study to provide an overview of the different formations and utilisation possibilities of IMCUs. Earlier studies on multiple IMCUs have so far focussed on the effect of the IMCU on the ICU mortality [7], ICU readmission rates and in-hospital mortality [1], cost-effectiveness of IMCUs [65], required staffing level at the IMCU [66], have specifically focussed on IMCUs designed for small spectrums function [67] or have described utilisation of IMCUs narratively [3].

In this study, the nomenclature of the IMCU was defined and a descriptive overview of the different utilisation of the IMCU with respect to admitting specialties, format, and location is provided. Through subsequently determining its common denominators in terms of possibilities and limitations, we hereby opened the door for further standardized research in a field where standardization is highly needed to support data comparison [3]. One of the main challenges will now be to identify the correct patient for safe admission at these different types of IMCUs. Another main challenge is to further explore the negative (and positive) consequences of IMCUs, such as delayed necessary ICU care and intubation.

The main limitation of our study was the probable publication bias. It is very likely that IMCUs with a successful IMCU did publish about their unit while those with an unsuccessful IMCU did not publish about their unit. Also, those who did publish about their IMCU were more likely to publish about their successes than their failures. These factors could have led to an overestimation of the possibilities of IMCUs and an underestimation of their limitations. However, thanks to this publication bias, excellent insight was provided in how an IMCU can be formatted, which modalities can be used, and how adequately functioning IMCUs differ from an ICU or ward.

Another limitation of our study was the reporting bias. In time, there have been only 47 articles describing an IMCU. Of these articles, most of them did not publish with the aim to describe their IMCU but instead performed other medical research at their IMCU and in publishing these results also described some characteristics of their IMCU. The information about their IMCU was therefore often incomplete. These missing data meant that our study very likely did not provide a complete overview of the formatting and possibilities of all the IMCUs reported. This also means that the found and reported significance levels would possibly not be significant if all studies had reported their data. Also, since we are confident that our search was comprehensive and complete, the relative small number of articles describing the IMCU reflects the need for additional research to this supportive unit.

## 5. Conclusion

This study defined the nomenclature of the IMCU and is the first to show the different formatting possibilities and supportive treatment modules of IMCUs. Furthermore, it shows that supportive modalities differ between IMCUs although all offer continuous monitoring and respiratory support while mechanical ventilation and the use of multiple vasoactive medications are limitations.

These findings are relevant to hospitals who seek to implement an IMCU. For hospitals who yet have an IMCU available, this study provides insights in to what extent (re)formatting options and supportive treatment modules could possibly be incorporated. Moreover, these findings open the door for future standardized research in this field.

## Supplementary Material

The Supplementary Material shows (1) a complete list of the search terms used and (2) the full study characteristics of the Intermediate Care Units included.

## Figures and Tables

**Table 1:** Summary of study characteristics of intermediate care units.

Authors, country of origin	Name of unit	Number of HDU beds	Location	Specialties	Format (O/C)	Patients per nurse
Armstrong et al. (2003) [21], Armstrong et al. (2015) [5], the Netherlands	MCU	9	I	Su, M	C	2.5
Bannister et al. (2016) [22], UK	HDU	2	W	Su	O	0.67
Batra et al. (2001) [23], UK	HDU	6	S	Su	O	x
Bellomo et al. (2005) [24], Australia	HDU	4	A	Su, M	C	2
Betten et al. (2016) [25], Norway	HDU	x	x	x	x	x
Coggins and de Cossart (1996) [26], Coggins and Infirmary (1998) [27], UK	HDU	6	x	Su	O	2
Confalonieri et al. (2015) [28], Italy	IMCU	15	x	M	x	4
Crosby and Rees (1983) [29], Crosby et al. (1990) [30]. UK	HDU	7	x	Su, M	O	2
Daud-Gallotti et al. (2012) [31], Ranzani et al. (2014) [32], Brazil	IMCU	11	S	Su, M	C	11
Davies et al. (1999) [33], UK	HDU	4	x	Su	O	x
Dhond et al. (1998) [34], UK	HDU	6	A	Su, M	C	x
Eachempati et al. (2004) [35], USA	SDU	4	A	Su	C	4
Edbrooke (1996) [36], UK	HDU	4	A	Su	C	x
Fox et al. (1999) [37], UK	HDU	4	A	X	x	2
Fujii et al. (2016) [38], Japan	IMCU	8	I	Su	C	x
Ghosh et al. 2004 [39], UK	HDU	6	S	Su	O	1.5
Gould et al. (2010) [40], Australia	HDU	8	A	Su, M	C	2
Harding (2009) [41], USA	IMCU	16	S	Su, M	O	3
Helm and Newman (1992) [42], UK	HDU	4	x	Su	O	2
Hilton et al. (1993) [43], USA	SDU	4	S	Su	O	2.5
Hravnak et al. (2008) [44], Hravnak et al. (2011) [45], Yousef et al. (2012) [46], USA	SDU	24	x	Su	x	6
Innocenti et al. (2014) [47], Italy	HDU	x	x	ED	C	x
Jones et al. (1992) [48], Jones et al. (1999) [49], UK	HDU	6	S	Su	O	2
Kalayi et al. (2001) [50], UK	HDU	4	x	Su	x	x
Keegan et al. (2008) [51], USA	PCU	x	S	Su	O	4
LeVasseur and Calder (1995) [52], Australia	HDU	4	x	Su	x	x
Lucena et al. (2012) [8], Lucena et al. (2013) [53], Alegre et al. (2015) [54], Martinez-Urbistondo et al. (2015) [55], Spain	IMCU	9	A	Su, M	C	3
Nehra et al. (1994) [56], UK	HDU	8	I	Su	x	2
Pilling et al. (2004) [57], UK	HDU	x	x	Su	O	x
Pirret (2002) [58], New Zealand	HDU	3	I	Su	O	3
Potena et al. (2004) [59], Italy	IMCU	x	x	M	x	x
Richards et al. (2012) [60], USA	IMCU	x	S	Su	O	x
Robertson et al. (2011) [19], UK	HDU	10	S	Su, M	O	x
Robertson et al. (2011) [19] UK^a^	x	10	S	Su, M	C	x
Shum et al. (2013) [61], Canada	SDU	x	W	Su, M	O	2
Solberg et al. (2008) [62], Solberg et al. (2014) [63], the Netherlands	IMCU	6	A	Su, M	C	3
Torres et al. (2006) [64], Spain	IMCU	20	A	Su, M	x	x
Yoo et al. (2015) [20], USA	PCU	10	A	M	C	3.5
Yoo et al. (2015) [20], USA^b^	x	15	S	M	O	3.5

^a^This Intermediate Care Unit was described in the same article as the one before, after changing its management format. ^b^This Intermediate Care Unit was described in the same article as the one before, after changing its location and management format.

This table shows a summary of the characteristics of the included Intermediate Care Units.

MCU = Medium Care Unit; HDU = High Dependency Unit; IMCU = Intermediate Care Unit; PCU = Progressive Care Unit; SDU = step-down unit; I = integrated in ICU; W = part of ward; A = adjacent to ICU; S = separate; Su = surgical patients; M = medical patients; O = open; C = closed.

## List of Abbreviations

IMCU: Intermediate Care Unit
ICU: Intensive Care Unit
TISS: Therapeutic Intervention Scoring System
NAS: Nursing Activities Score.
