# Supplementary material for: Utilisation of Intermediate Care Units: A Systematic Review
Source: Crit Care Res Pract. 2017 Jul 9;2017:8038460. doi: 10.1155/2017/8038460 (PMC5523340; doi:10.1155/2017/8038460)
Supplement: Supplementary file 1 — The Supplementary Material shows (1) a complete list of the search terms used and (2) the full study characteristics of the Intermediate Care Units included. [file 8038460.f1.pdf]

Supplementary Material 1. Search terms

(Medium Care Unit*[title/abstract] OR

Medium Medical Care Unit*[title/abstract] OR

Intermediate Care Unit*[title/abstract] OR

Intermediate Medical Care Unit*[title/abstract] OR

Medical Intermediate Care Unit*[title/abstract] OR

High Care Unit*[title/abstract] OR

High Medical Care Unit*[title/abstract] OR

High Dependency Unit*[title/abstract] OR

High-Dependency Unit*[title/abstract] OR

Progressive care unit*[title/abstract] OR

Step up/down unit*[title/abstract] OR

Step-up unit*[title/abstract] OR

Step up unit*[title/abstract] OR

Step-down unit*[title/abstract] OR

Step down unit*[title/abstract] OR

Transitional care unit*[title/abstract] OR

Medium Care Ward*[title/abstract] OR

Medium Medical Care Ward*[title/abstract] OR

Intermediate Care Ward*[title/abstract] OR

Intermediate Medical Care Ward*[title/abstract] OR

Medical Intermediate Care Ward*[title/abstract] OR

High Care Ward*[title/abstract] OR

High Medical Care Ward*[title/abstract] OR

High Dependency Ward*[title/abstract] OR

High-Dependency Ward*[title/abstract] OR

Progressive care Ward*[title/abstract] OR

Step up/down Ward*[title/abstract] OR

Step-up Ward*[title/abstract] OR

Step up Ward*[title/abstract] OR

Step-down Ward*[title/abstract] OR

Step down Ward*[title/abstract] OR

Transitional care Ward*[title/abstract])

NOT (

Child*[title/abstract] OR

Paediatric*[title/abstract] OR

Infant*[title/abstract] OR

Neonate*[title/abstract] OR

Pediatric*[title/abstract])

These terms were used in Medline, Embase & Cochrane

Supplementary Material 2. Full Study Characteristics of Intermediate Care Units

Authors, country of origin	Name of unit	# of HDU beds	Location	Specialties	Format (O/C)	Patients per nurse	Hemodynamic monitoring	Invasive monitoring	Respiratory support	Mechanical ventilation (weaning)	High-flow oxygen therapy	NIV/CPAP	Tracheostomy care	Single vasoactive medication	Multiple vasoactive medication	Sedatives	Intracranial pressure	Renal Replacement Therapy	Specific interventions	Residents	# of Residents	Registrars	# of Registrars	Consultants	# of Consultants
Armstrong et al. (2003) [21], Armstrong et al. (2015) [5], the Netherlands	MCU	9	I	Su, M	C	2.5	1	1	1	x	x	1	x	1	x	1	1	x	x	x	x	x	x	x	x
Bannister et al. (2016) [22], UK	HDU	2	W	Su	O	0.67	1	x	1	x	x	x	x	x	x	x	x	x	x	x	x	x	x	x	x
Batra et al. (2001) [23], UK	HDU	6	S	Su	O	x	1	x	x	x	x	x	x	x	x	x	x	x	x	x	x	x	x	1	x
Bellomo et al. (2005) [24], Australia	HDU	4	A	Su, M	C	2	1	x	1	0	x	1	x	1	x	x	x	0	x	1	x	1	x	1	x
Betten et al. (2016) [25], Norway	HDU	x	x	x	x	x	x	x	1	x	x	x	x	1	x	x	x	x	x	x	x	x	x	x	x
Coggins and de Cossart (1996) [26], Coggins and Infirmary (1998) [27], UK	HDU	6	x	Su	O	2	1	1	1	0	x	1	1	1	x	x	x	0	1	1	1	x	x	x	x
Confalonieri et al. (2015) [28], Italy	IMCU	15	x	M	x	4	x	x	x	x	x	x	x	x	x	x	x	x	x	x	x	x	x	1	1
Crosby and Rees (1983) [29], Crosby et al. (1990) [30]. UK	HDU	7	x	Su, M	O	2	1	x	x	x	x	x	x	x	x	x	x	x	x	x	x	x	x	x	x
Daud-Gallotti et al. (2012) [31], Ranzani et al. (2014) [32], Brazil	IMCU	11	S	Su, M	C	11	1	x	1	0	0	1	x	0	0	x	x	1	x	1	2	x	x	1	1
Davies et al. (1999) [33], UK	HDU	4	x	Su	O	x	1	1	x	0	x	0	x	0	0	x	x	x	x	x	x	x	x	x	x
Dhond et al. (1998) [34], UK	HDU	6	A	Su, M	C	x	x	x	x	x	x	x	x	x	x	x	x	x	x	x	x	x	x	x	x
Eachempati et al. (2004) [35], USA	SDU	4	A	Su	C	4	1	x	x	1	x	x	x	x	x	x	x	x	x	1	x	x	x	1	1
Edbrooke (1996) [36], UK	HDU	4	A	Su	C	x	x	x	x	x	x	x	x	x	x	x	x	x	x	x	x	x	x	x	x
Fox et al. (1999) [37], UK	HDU	4	A	X	x	2	x	x	x	x	x	x	x	x	x	x	x	x	x	x	x	x	x	x	x
Fujii et al. (2016) [38], Japan	IMCU	8	I	Su	C	x	1	1	x	1	x	1	x	1	x	x	x	x	x	x	x	x	x	x	x
Ghosh et al. 2004 [39], UK	HDU	6	S	Su	O	1.5	1	1	x	0	x	1	x	0	0	x	x	x	x	x	x	x	x	x	x
Gould et al. (2010) [40], Australia	HDU	8	A	Su, M	C	2	1	1	x	x	x	1	x	1	x	x	0	0	x	1	1	x	x	1	1
Harding (2009) [41], USA	IMCU	16	S	Su, M	O	3	x	x	x	1	x	x	x	x	x	x	x	x	x	x	x	x	x	x	x
Helm and Newman (1992) [42], UK	HDU	4	x	Su	O	2	1	1	x	0	x	0	x	x	x	x	x	x	x	x	x	x	x	x	x
Hilton et al. (1993) [43], USA	SDU	4	S	Su	O	2.5	x	x	x	x	x	x	x	x	x	x	x	x	x	x	x	x	x	x	x

Definition of abbreviations: MCU=Medium Care Unit; HDU=High Dependency Unit; IMCU=Intermediate Care Unit; PCU=Progressive Care Unit; SDU=Step Down Unit; I= integrated in ICU; W=part of Ward; A=Adjacent to ICU; S=Separate; Su=surgical patients; M=medical patients; O=Open; C=Closed; NIV=Non-Invasive Ventilation; CPAP=Continuous Positive Airway Pressure

Authors, country of origin	Name of unit	# of HDU beds	Location	Specialties	Format (O/C)	Patients per nurse	Hemodynamic monitoring	Invasive monitoring	Respiratory support	Mechanical ventilation (weaning)	High-flow oxygen therapy	NIV/CPAP	Tracheostomy care	Single vasoactive medication	Multiple vasoactive medication	Sedatives	Intracranial pressure	Renal Replacement Therapy	Specific interventions	Residents	# of Residents	Registrars	# of Registrars	Consultants	# of Consultants
Hravnak et al. (2008) [44], Hravnak et al. (2011) [45], Yousef et al. (2012) [46], USA	SDU	24	x	Su	x	6	1	x	x	x	x	x	x	x	x	x	x	x	x	x	x	x	x	x	x
Innocenti et al. (2014) [47], Italy	HDU	x	x	ED	C	x	1	1	x	0	x	x	x	1	x	x	x	x	x	x	x	x	x	x	x
Jones et al. (1992) [48], Jones et al. (1999) [49], UK	HDU	6	S	Su	O	2	1	x	x	0	x	x	x	x	x	x	x	x	x	x	x	1	1	1	x
Kalayi et al. (2001) [50], UK	HDU	4	x	Su	x	x	x	x	x	x	x	x	x	x	x	x	x	x	x	x	x	1	1	x	x
Keegan et al. (2008) [51], USA	PCU	x	S	Su	O	4	1	x	x	0	x	1	x	0	0	x	x	x	x	x	x	x	x	x	x
LeVasseur and Calder (1995) [52], Australia	HDU	4	x	Su	x	x	1	1	x	x	x	x	x	x	x	x	x	x	x	x	x	x	x	x	x
Lucena et al. (2012) [8], Lucena et al. (2013) [53], Alegre et al. (2015) [54], Martinez-Urbistondo et al. (2015) [55], Spain	IMCU	9	A	Su, M	C	3	1	1	x	x	x	1	x	x	x	x	x	x	x	1	1	x	x	1	1
Nehra et al. (1994) [56], UK	HDU	8	I	Su	x	2	x	x	x	x	x	x	x	x	x	x	x	x	x	x	x	x	x	x	x
Pilling et al. (2004) [57], UK	HDU	x	x	Su	O	x	1	1	x	0	x	1	x	1	0	x	x	0	x	x	x	x	x	x	x
Pirret (2002) [58], New Zealand	HDU	3	I	Su	O	3	1	1	1	x	1	1	x	1	x	x	x	x	x	x	x	x	x	x	x
Potena et al. (2004) [59], Italy	IMCU	x	x	M	x	x	1	0	x	1	x	1	1	x	x	x	x	x	x	x	x	x	x	x	x
Richards et al. (2012) [60], USA	IMCU	x	S	Su	O	x	x	x	x	x	x	x	x	x	x	x	x	x	x	x	x	x	x	x	x
Robertson et al. (2011) [19], UK	HDU	10	S	Su, M	O	x	1	x	x	x	x	1	x	x	x	x	x	1	x	x	x	x	x	x	x
Robertson et al. (2011) [19] UK	x	10	S	Su, M	C	x	1	x	x	x	x	1	x	x	x	x	x	1	x	x	x	x	x	x	x
Shum et al. (2013) [61], Canada	SDU	x	W	Su, M	O	2	1	1	x	0	x	1	x	1	x	x	0	0	x	x	x	x	x	x	x
Solberg et al. (2008) [62], Solberg et al. (2014) [63], the Netherlands	IMCU	6	A	Su, M	C	3	x	x	x	x	x	x	x	x	x	x	x	x	x	x	x	x	x	x	x
Torres et al. (2006) [64], Spain	IMCU	20	A	Su, M	x	x	1	1	x	1	x	1	x	1	x	x	x	1	x	x	x	x	x	x	x
Yoo et al. (2015) [20], USA	PCU	10	A	M	C	3.5	1	0	x	1	x	x	x	x	x	x	0	x	x	1	x	x	x	1	x
Yoo et al. (2015) [20], USA	x	15	S	M	O	3.5	1	0	x	1	x	x	x	x	x	x	0	x	x	x	x	x	x	x	x

[a]This Intermediate Care Unit was described in the same article as the one above, after changing its management format.
[b]This Intermediate Care Unit was described in the same article as the one above, after changing its location and management format.

Definition of abbreviations: MCU=Medium Care Unit; HDU=High Dependency Unit; IMCU=Intermediate Care Unit; PCU=Progressive Care Unit; SDU=Step Down Unit; I= integrated in ICU; W=part of Ward; A=Adjacent to ICU; S=Separate; Su=surgical patients; M=medical patients; O=Open; C=Closed; NIV=Non-Invasive Ventilation; CPAP=Continuous Positive Airway Pressure
